# Current status of development of methylation biomarkers for in vitro diagnostic IVD applications

**DOI:** 10.1186/s13148-020-00886-6

**Published:** 2020-07-06

**Authors:** Olga Taryma-Leśniak, Katarzyna Ewa Sokolowska, Tomasz Kazimierz Wojdacz

**Affiliations:** Independent Clinical Epigenetics Laboratory, Pomeranian Medical University, Unii Lubelskiej 1, 71-252 Szczecin, Poland

**Keywords:** DNA methylation, IVD, In Vitro diagnostics, Biomarker, Epigenetics

## Abstract

A significant volume of research clearly shows that disease-related methylation changes can be used as biomarkers at all stages of clinical disease management, including risk assessment and predisposition screening through early diagnostics to personalization of patient care and monitoring of the relapse and chronic disease. Thus disease-related methylation changes are an attractive source of the biomarkers that can have significant impact on precision medicine. However, the translation of the research findings in methylation biomarkers field to clinical practice is at the very least not satisfactory. That is mainly because the evidence generated in research studies indicating the utility of the disease-related methylation change to predict clinical outcome is in majority of the cases not sufficient to postulate the diagnostic use of the biomarker. The research studies need to be followed by well-designed and systematic investigations of clinical utility of the biomarker that produce data of sufficient quality to meet regulatory approval for the test to be used to make clinically valid decision. In this review, we describe methylation-based IVD tests currently approved for IVD use or at the advanced stages of the development for the diagnostic use. For each of those tests, we analyze the technologies that the test utilizes for methylation detection as well as describe the types of the clinical studies that were performed to show clinical validity of the test and warrant regulatory approval. The examples reviewed here should help with planning of clinical investigations and delivery of the clinical evidence required for the regulatory approval of potential methylation biomarker based IVD tests.

## Epigenetic biomarkers in precision medicine

Covalent addition of methyl group (-CH_3_) to the 5-carbon position of the cytosine is one of the first epigenetic modifications shown to modulate gene expression regulation. In general terms, methylation of cytosines in the gene promoter interferers with the gene transcription and can lead to transcriptional gene deactivation. Throughout the decades of research, a number of other than DNA methylation mechanisms of the epigenetic gene expression regulation have been discovered. All of those mechanisms interconnect to orchestrate the expression of specific genes at a specific time to provide specific cell phenotype. (The description of different levels of epigenetic gene regulation is out of scope of this review and can be found in for example [1–3]).

In medicine, a biomarker is any measurable indicator of a particular disease or physiological state of an organism. Thus, any epigenetic modification of DNA, RNA, or protein that induces gene expression change which in turn results in a specific phenotype is a biomarker of that phenotype. However, currently, only DNA methylation seems to be sufficiently stable epigenetic modification to be utilized as a biomarker in in vitro diagnostic (IVD) settings. This modification is not only stable in somatic cells and is populated to daughter cells with high fidelity, but more importantly from the in vitro diagnostic applicability prospective, DNA methylation has been shown not to be affected by sample processing and storage conditions of the clinical material [4]. Those features make disease-related methylation changes an attractive source of the IVD biomarkers.

**Fig. 1:**
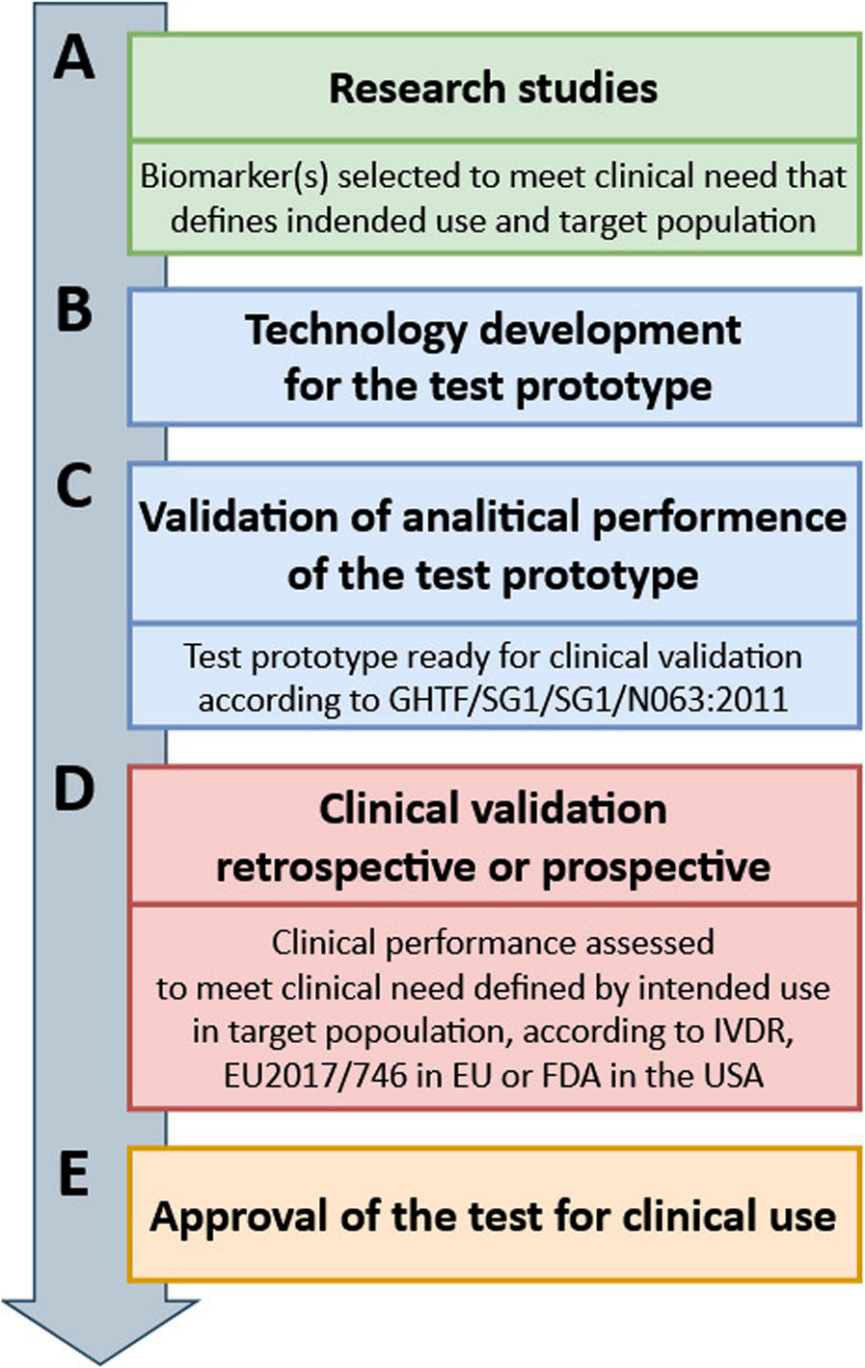
Overview of the process of in vitro diagnostic (IVD) test development for clinical use. Parts **a**-**e** show stages of the test development from research studies (**a**) to clinical use (**e**), through technology development (**b**), analitical validation (**c**) and clinical validation (**d**)

There is already vast research evidence available, showing that disease-related methylation changes can be utilized as biomarkers in the clinical management of various diseases. That research also indicates that potential fields of applications of methylation biomarkers include risk assessment, diagnosis, treatment management, and post-treatment monitoring. However, the results of the research studies that lead to biomarker discovery and generate an evidence for the clinical utility of the biomarker are almost never sufficient to postulate the diagnostic use of the biomarker. Nevertheless, those studies allow to define intended use of the test which describes the clinical need addressed by the prospective biomarker testing as well as target population which is a patient group subjected to testing (Fig. 1a). Clear definition of the intended use of and target population are critical for the recruitment of the specific patient group for clinical validation studies which in principle, are well-controlled and systematic investigations aiming to show clear benefit and safety of use of the biomarker in clinical practice (Fig. 1d). Overall, clinical validation studies need to provide evidence that the biomarker testing meets clinical need defined by intended use in the target population at the quality that fulfills the regulatory requirements for the approval of the test for the IVD use. Clinical validation studies also require a prototype of the prospective IVD test with validated analytical performance parameters (Fig. 1b, c).

## Components of the in vitro diagnostic test components

In the process of development of the IVD test, the biomarker (here disease related methylation change) is considered only one of two components of the IVD test. The use of a biomarker in routine diagnostic practice requires a second component, namely the technology enabling the detection of the biomarker in the clinical material (Fig. 1b). The performance of the technology to detect biomarker is assessed during pre-clinical, analytical test validation which results in development of the prototype test (Fig. 1c). This procedure includes an examination of the test characteristics such as analytical sensitivity (attributed by, e.g., limit of blank, limit of detection, and limit of quantitation), analytical specificity (the ability of the method to determine solely targeted marker), accuracy of measurement (derived from trueness—systemic error, and precision—random error), measuring range (linear and non-linear measuring systems), and the definition of cut-off value for the measurement the assay provides, as described in Global Harmonization Task Force document GHTF/SG1/SG1/N063:2011.

The prototype needs to undergo clinical validation (Fig. 1d) before it can be marketed as IVD test. The clinical validation is a study designed to show how accurately potential IVD test predicts the risk for, or presence of a given clinical condition. The accuracy of the test is measured with diagnostic (clinical) sensitivity and specificity. The diagnostic sensitivity is a probability of the test to give a positive result in the presence of targeted marker, while diagnostic specificity is a probability of the test to give a negative result in the absence of the targeted marker, as defined in Commission Decision of 27 November 2009 (nr 2009/886/EC). The clinical validation study can be retrospective or prospective and it always aims to show the clinical benefit of the use of test in relation to the present test or practice. Overall, this type of study needs to generate data of sufficient quality for the prospective biomarker to meet requirements of In Vitro Diagnostic Device Regulation (IVDR, EU 2017/746) in the European Union, to obtain Conformité Européenne (CE) marking, and procedures of Food and Drug Administration (FDA) approval in the USA. Only meeting those requirements permits the use of the IVD test (Fig. 1e). Currently, there are no IVD tests targeting methylation changes outside of oncology, thus in subsequent sections, we review biomarkers that are already approved or are at the advanced stages of development for the IVD use in clinical cancer management.

## In vitro diagnostic use of the methylation-based biomarkers

As already mentioned, the fields of use of the IVD tests in clinical disease management can, for example, be subdivided into four areas representing natural stages of the disease development and including the disease risk assessment—before the disease develops, diagnosis—when disease is already present, treatment personalization—while treating the disease, and as increasing number of the diseases especially cancers become chronic disease—post-treatment monitoring/surveillance. The aim of this review is to describe current applications of diagnostic tests targeting methylation biomarkers in each of those fields of use. Table 1 provides overview of all the tests that we were able to identify and the following text contains detailed description of the test and the clinical evidence supporting diagnostic use of the test.

**Table 1:** Commercially available IVD tests by the type of methylation-based biomarker use

Test name	Type of cancer	Methylation biomarker	Detection technology	Biosample (as referenced by manufacturer)	Manufacturer/Distributor	Type of approval (year)
Diagnostic biomarkers						
Cologuard®	Colorectal cancer	*BMP3, NDRG4* (+ *KRAS* seven point mutations)	QuARTS^TM^	Stool	Exact Sciences Co., Madison, WI, USA	FDA (2014)
Epi proColon®	Colorectal cancer	*SEPT9*	Real-time PCR with fluorescent hydrolysis probe	Plasma	Epigenomics AG, Berlin, Germany	FDA (2016)
Real Time mS9 CRC Assay	Colorectal cancer	*SEPT9*	Real-time PCR	Plasma	Abbott Laboratories, Chicago, IL, USA	CE (2010)
EarlyTect® Colon Cancer	Colorectal cancer	*SDC2*	Qualitative methylation-specific real-time PCR	Stool	Genomictree Inc., Daejeon, South Korea	CE (2017)
HCCBloodTest	Hepatocellular carcinoma	*SEPT9*	Real-time PCR with fluorescent hydrolysis probe	Plasma	Epigenomics AG, Germany	CE (2019)
No commercial name available	Hepatocellular carcinoma	*DAB2IP*, *EMX1*, *HOXA1, TSPYL5* (+ two proteins: AFP and lectin bound AFP)	QuARTS^TM^	Blood	Exact Sciences Co., Madison, WI, USA	Breakthrough Device designation (2019)
IvyGene Dx Liver Cancer Test	Hepatocellular carcinoma	Not provided	Targeted next-generation sequencing	Plasma	Laboratory for Advanced Medicine Inc., Irvine, CA, USA	Breakthrough Device designation (2019)
Epi proLung®	Lung cancer	*SHOX2, PTGER4*	Real-time PCR with fluorescent hydrolysis probe	Plasma	Epigenomics AG, Berlin, Germany	CE (2017)
AssureMDx	Bladder cancer	*OTX1*, *ONECUT2*, *TWIST1* (+ mutations in: *FGFR3*, *TERT*, *HRAS*)	Multiplex SNaPshot® assay	Voided urine	MDxHealth, Irvine, CA, USA	LDT (2017)
Bladder *CARE*™	Bladder cancer	*SOX1*, *IRAK3*, *LINE1*	MSRE-qPCR	Voided urine	Pangea Laboratory, Costa Mesa, CA, USA	LDT (2019)
ConfirmMDx	Prostate cancer	*GSTP1*, *RASSF1*, *APC*	Multiplexed quantitative DNA methylation-specific PCR	Prostate biopsy	MDxHealth, Irvine, CA, USA	LDT (2012)
GynTect®	Cervical cancer	*ASTN1*, *DLX1*, *ITGA4*, *RXFP3*, *SOX17*, *ZNF671*	Methylation-specific real-time PCR	Cervical smear	Oncognostic GmbH, Jena, Germany	CE (2019)
QIAsure Methylation Test Kit	Cervical cancer	*FAM19A4, MIR124-2*	Multiplex real-time PCR	Cervical/vaginal specimens	QIAGEN GmbH, Hilden, Germany	CE (2016)
PAX1 DNA Detection Kit	Cervical cancer	*PAX1*	Real-time PCR	Cervical/oral scrapes	iStat Biomedical Co. Ltd., New Taipei City, Taiwan	CE (2016)
ZNF582 DNA Detection Kit	Cervical cancer	*ZNF582*	Real-time PCR	Cervical/oral scrapes	iStat Biomedical Co. Ltd., New Taipei City, Taiwan	CE (2016)
GRAIL	Cancer regardless of its type	Multiple CpG sites	Whole-genome bisulfite sequencing	Blood	GRAIL Inc., Menlo Park, California, USA	Breakthrough Device designation (2019)
IvyGene® Cancer Blood Test	Cancer regardless of its type	*MYO1G, TNFAIP8L2*	Targeted next-generation sequencing	Plasma	Laboratory for Advanced Medicine Inc., Irvine, CA, USA	LDT (2018)
Disease management biomarkers						
*therascreen*® MGMT Pyro® Kit	Glioblastoma	*MGMT*	Pyrosequencing®	FFPE tumor tissue	QIAGEN GmbH, Hilden, Germany	CE (2012)
Human MGMT Gene Methylation Detection Kit	Glioblastoma	*MGMT*	PAP-ARMS®	FFPE tumor tissue	Xiamen SpacegenCo., Ltd., Xiamen, China	CE (2016)
MGMT Methylation Detection Kit	Glioblastoma	*MGMT*	Real-time PCR with fluorescent hydrolysis probes and methylation-specific primers	Fresh frozen or FFPE tumor tissue	EntroGen, Inc., Los Angeles, CA, USA	CE (2018)
PredictMDx	Glioblastoma	*MGMT*	Quantitative methylation-specific PCR	FFPE tumor tissue	LabCorp, Burlington, NC, USA	LDT (2012)
therascreen® PITX2 RGQ PCR Kit	Breast cancer	*PITX2*	Real-time PCR with fluorescent hydrolysis probes and methylation-unspecific primers	FFPE tumor tissue	QIAGEN GmbH, Hilden, Germany	CE (2018)
EPICUP®	Cancers of unknown primary site	Multiple CpG sites	MethylationEPIC 850K array (Illumina, San Diego, CA, USA)	Fresh frozen or FFPE tumor biopsy	Grupo Ferrer Internacional SA, Barcelona, Spain	CE (2015)
Post-treatment monitoring biomarkers						
COLVERA^TM^	Colorectal cancer	*IZKF1, BCAT1*	Multiplex real-time PCR	Plasma	Clinical Genomics PathologyInc, Bridgewater, NJ, USA	LDT (2016)
Bladder EpiCheck®	Bladder cancer	15 methylation biomarkers*	MSRE-qPCR	Voided urine	Nucleix Ltd., Rehovot, Israel	CE (2017)

*QuARTS*^TM^ quantitative allele-specific real-time target and signal amplification, *MSRE*-*qPCR* methylation-sensitive restriction enzyme followed by real-time PCR, *PAP*-*ARMS*® technology combination of pyrophosphorolysis-activated polymerization reaction (PAP) and amplification refractive mutation system (ARMS)

*Patent application nr US9458503B2

### Risk assessment biomarkers

It is well established that environmental exposure can induce methylation changes that may lead to adverse health effects. Smoking is one of the best-researched example of such a type of the exposure. A recent meta-analysis reviewed the evidence for smoking to induce methylation changes in blood cells of children whose mothers smoked during pregnancy. The analysis included 13 studies with nearly 7000 of new-born and five studies with over 3000 of older children (average age = 6.8 years) and identified over 6000 differentially methylated CpG sites in the blood of both, new-born and older children of smoking mothers. Furthermore, a number of identified changes was associated with the changes of gene expression. The analysis of the functional context of these changes showed that they may affect pathways critical in development and the results were concordant in both children groups. It is worth mentioning that the top hit with the lowest *p* value in this study was CpG site located in the *AHRR* gene [5] and methylation changes at this CpG site were identified in many other studies involving smoking adults [6–9] and children of smoking mothers [10–12]. Considering the magnitude of the evidence, the methylation changes at the CpG site in *AHRR* can already be proposed as a biomarker of exposure to smoking and adverse smoking-related health effects even in former smokers.

Occupational agents have also been shown to induce methylation changes and biomarkers enabling detection of the effects of those exposures are especially important in the context of the work environment regulations. For example, trichloroethylene (TCE) is a volatile and colorless liquid that among other applications is used in some household products, such as cleaning wipes, aerosol cleaning products, tool cleaners, paint removers, spray adhesives, carpet cleaners, and spot removers. The wide use of this substance makes it an occupational toxicant for various working groups. Moreover, TCE exposure has already been associated with kidney cancer [13–16], autoimmune diseases [17], and non-Hodgkin lymphoma [18–20]. Recent, epigenome-wide association study that included 37 and 30 workers exposed to high and low levels of this substance, respectively, and 73 unexposed controls identified 25 CpG sites with TCE-related methylation changes and a region in the promoter of *TRIM68* gene that displayed hypomethylation with increased exposure to TCE. The analyses of the functional context of the identified changes linked them to pathways important in the development of autoimmune diseases; and in genes related to cancer development [21].

Lead (Pb) exposure has been reported to have adverse effect mainly on nervous, hemopoietic, and renal systems [22]. A recent genome-wide analysis identified 354 CpG sites differently methylated between individuals with low and high lead exposure. The study was based on only four cases in each of the exposure groups but the results were validated in independent cohort of 15 cases in low and 15 in high exposure groups. Moreover, two of the differently methylated CpG sites within *GSTM1* gene identified in this study have been previously shown to correlate with Pb exposure [23, 24]; other methylation changes were linked to *CRIM1* and *NINJ2* genes, which are important regulatory genes of nervous system development [25].

Exposure to welding fumes is a serious occupational challenge mainly due to the size of the welding industry. Epidemiological studies identified the metal-rich mixture of fine and ultrafine particulate matter (PM) generated by welding in extreme heat conditions cause respiratory health effects, such as lung function changes, asthma, bronchitis, and cardiovascular diseases. Moreover, welding fumes are classified in the first group of carcinogens to humans (IARC 22nd citation from this article). V. Leso et al. have recently reviewed evidence for this exposure to induce methylation changes and concluded that although it is still not clear how lead exposure-related methylation changes link to the adverse health effects, each of the studies analyzed presented strong evidence for welding fumes exposure to induce methylation changes in exposed individuals [26].

Natural pollutants are another example of the environmental exposure that can present a serious health risk in some geographical regions. For example, inorganic arsenic (iAs) is a relatively well-studded environmental pollutant present in water and certain food in specific geological regions and exposure to arsenic is a risk factor for different types of cancers as well as cardiovascular and lung diseases [27]. We have shown that exposure to this pollutant induced methylation changes in CD4^+^ cells that can potentially affect the immune system [28]. Others showed significant associations between gene-specific methylation changes in the DNA from blood cells and arsenic exposure [29]. Those findings suggest that epigenetic modifications may be an important element of mechanism underlying arsenic toxicity and thus biomarkers of that exposure.

In conclusion, the link between methylation changes induced by environmental factors and specific health effects is still not clear and needs to be further studied. However, the above examples clearly indicate the potential applicability of the environmentally induced methylation changes as a risk assessment biomarker.

### Diagnostic biomarkers

As a general paradigm, early detection of the disease significantly increases the chance of cure. The studies of carcinogenesis of different types of cancers as well as pathogenesis of other diseases show that methylation changes occur early in the disease development [30–32]. It is also well established that the DNA from pathologically changed cells is secreted and can be detected in body fluids such as sputum, plasma, urine, or stool. Those body fluids are referred to as liquid biopsies. In most cases, liquid biopsies can be obtained with none or minimal invasiveness which opens a convenient way for the early disease diagnosis.

#### Colorectal cancer

Colorectal cancer (CRC) is the third most common cancer and the fourth leading cause of cancer-related deaths worldwide [33]. Five-year survival rates in this cancer drop drastically from 90 to 10% with increasing stage at the diagnosis. Currently, FDA-approved Cologuard® (Exact Sciences Co., Madison, WI, USA) and Epi proColon® (Epigenomics AG, Berlin, Germany) and CE-marked RealTime mS9 CRC Assay (Abbott, Chicago, IL, USA) and EarlyTect® Colon Cancer (Genomictree Inc., Daejeon, South Korea) are liquid biopsy-based tests targeting methylation changes intended for CRC screening.

Cologuard® is the multi-target stool DNA test, intended to CRC screening in adults of 45 or older, who are at typical average-risk for CRC. The test is recommended to be performed every 3 years and targets methylation changes at *BMP3* and *NDRG4* promoters and seven, point mutations in the *KRAS* gene*.* The assay is built on QuARTS^TM^ technology [34] and also requires immunochemical assessment for the stool hemoglobin [35]. Initial validation of the test was performed in a multicenter study (Clinical Trial NCT01397747) on a group of 9989 adults showing sensitivity of 92% (95% confidence interval (95% CI) 83.0–97.5), and specificity of 87% (95% CI 85.9–87.2) for detection of CRC and 42% (95% CI 38.9–46.0) for detection of advanced adenomas [35]. The performance of the test was further evaluated in the prospective clinical trial (NCT02419716) that recruited 1119 subjects, 50 years and older and retrospective clinical trial (NCT03705013) performed on four sub-populations (positive first Cologuard® test; negative colonoscopy or no colonoscopy; positive 3-year follow-up Cologuard® test; negative colonoscopy or no colonoscopy) from the initially recruited cohort. The post-market clinical validation of the test is still ongoing in a prospective clinical trial (NCT04124406), aiming to recruit 150,000 participants, 18 years and older.

Epi proColon® is also a screening test, intended for adults, 50 years or older, with average risk for CRC, who have been offered and have a history of not completing CRC screening. The test is built on a real-time PCR technology with a fluorescent hydrolysis probe and targets the methylation changes of the *SEPT9* gene promoter in cell-free DNA (cfDNA) extracted from plasma. The initial assessment of the clinical performance of the Epi proColon® was performed in a prospective multicenter study (Clinical Trial NCT00855348) that included 53 CRC cases and 1457 subjects without CRC. The study showed sensitivity of the test at the level of 48.2% (95% CI 32.4–63.6, crude rate 50.9%) and specificity of 91.5% (95% CI 89.7–93.1, crude rate 91.4%) for CRC detection, as well as a sensitivity of 11.2% for the detection of adenomas [36]. A subsequent study, comparing the performance of Epi proColon® with an established fecal immunochemical test (FIT) that uses antibodies to detect blood in the stool as a symptom of the CRC (Clinical Trial NCT01580540), showed the Epi proColon sensitivity of 73.3% (95% CI 63.9–80.9) and specificity of 81.5% (95% CI 75.5–86.3), compared with 68.0% (95% CI 58.2–76.5) and 97.4% (95% CI 94.1-98.9), respectively for FIT [37]. That indicates a good performance of the test but the study by Ørntoft et al. [38] reported that the performance of the Epi proColon test is negatively affected by factors commonly associated with CRC screening populations including early-stage disease (low sensitivity in detecting adenomas and stage I carcinomas), diabetes, arthritis, arteriosclerosis, and age > 65 years. Since 2011, the second generation of Epi proColon® 2.0 CE is marketed in EU and Asian Pacific regions. The clinical validation of this version of the test was performed using plasma samples from 92 patients with no evidence of disease before colonoscopy (controls) and 92 patients with CRC before surgical treatment. The study reported the test sensitivity to detect CRC at the level of 79.3% (95% CI 69.6–87.1) and specificity of 98.9% (95% CI 94.1–100). Additionally, the study compared Epi proColon® 2.0 CE test performance with the standard guaiac-based fecal occult blood test (gFOBT) and a serum-based tumor marker for CRC—carcinoembryonic antigen (CEA). The gFOBT (17 controls, 22 CRC) showed 68.2% (95% CI 45.1–86.1) sensitivity and 70.6% (95% CI 44–89.7) specificity to detect CRC. The sensitivity and specificity of CEA test (27 controls, 27 CRC) for CRC detection was 51.8% (95% CI 31.9–71.3) and 85.2% (95% CI 66.3–95.8), respectively [39]. Another study evaluating the clinical performance of Epi proColon® 2.0 CE assay included 135 patients with CRC, 169 with adenomatous polyps, 81 with hyperplastic polyps, and 91 controls. The results of the study reported the test sensitivity for the CRC detection at the level of 74.8% (95% CI 67.0–81.6) and specificity of 87.4% (95% CI 83.5–90.6). Moreover, this study compared the performance of Epi proColon® 2.0 CE assay with the FIT test and showed FIT sensitivity to detect CRC at the level of 58.0% (95% CI 46.1–69.2) and specificity at the level of 82.4% (95% CI 74.4–88.7) [40]. The Epi proColon® is now being postmarket validated in a longitudinal prospective clinical trial (NCT03218423), aiming to enroll 4500 participants, 50 to 74 years old.

It is worth mentioning that Abbott Laboratories (Chicago, IL, USA) developed its own version of the test targeting the same biomarker as Epi proColon® (*SEPT9*) and is marketing it under the name RealTime mS9 CRC Assay. The test is intended for CRC detection in cfDNA extracted from plasma, using real-time PCR technology and is not approved by the FDA but CE-marked under the EU directive (98/79/EC).

EarlyTect® Colon Cancer test is also CE-marked and targets *SDC2* gene methylation in DNA extracted from stool, using qualitative methylation-specific real-time PCR technology. The test was initially developed for the assessment of *SDC2* methylation in cfDNA from serum. The study performed using serum samples from 131 CRC patients at stages I to IV and 125 healthy individuals, demonstrated the biomarker sensitivity of 87.0% (95% CI 80.0–92.3) to detect CRC stages I to IV, with a specificity of 95.2% (95% CI 89.8–98.2) [41]. However, the test was finally developed for *SDC2* methylation analysis in DNA extracted from stool of CRC patients. The performance of EarlyTect® Colon Cancer test was evaluated in the study (Clinical Trial NCT03146520) that included the group of 585 evaluated subjects (245 with CRC, 44 with various sized adenomatous polyps, and 245 with negative colonoscopy results) and showed overall sensitivity of the test to detect CRC at the level of 90.2% (95% CI 85.8–93.6) with area under the curve (AUC) of 0.902 (95% CI 0.876–0.928), regardless of tumor stage, location, sex, or age (*p* > 0.05), and the specificity of 90.2% (95% CI 85.8–93.6) [42].

#### Hepatocellular carcinoma

Hepatocellular carcinoma (HCC) is the sixth most common cancer and the second most frequent cause of cancer death worldwide [33]. As described in the above, Epi proColon® was originally developed for the detection of CRC but was also evaluated in the context of HCC diagnosis. The clinical utility of the Epi proColon® 2.0 CE test to detect HCC was initially performed in the study that included 289 patients with cirrhosis, among whom 98 had HCC. The results showed that the test was able to detect HCC with a sensitivity of 90.6% (95% CI 81.9–99.2) and the specificity of 87.2% (95% CI 80.8–93.7) which is significantly higher than the accuracy of alpha-fetoprotein (AFP), widely used serum diagnostic marker for liver cancer (difference between area under the receiver operating characteristics (AUROCs) at the level of 0.115 (95% CI 0.042–0.187), standard error (SE) = 0.04, *p* = 0.002) [43]. The Epi proColon® 2.0 CE test for HCC detection is now evaluated in ongoing prospective clinical trial (NCT03311152) aiming to recruit 440 patients with cirrhosis. Epigenomics AG (Berlin, Germany), the manufacturer of the is Epi proColon® 2.0 CE, is now most likely marketing this test under the name HCCBloodTest as a CE-marked, intended to detect HCC in patients with cirrhosis. However, we were not able to confirm that both tests utilize the same assay. The performance of the test is now validated in the ongoing clinical trial (NCT03804593).

Recently, Exact Sciences Co. (Madison, WI, USA) released promising data describing performance of an early HCC detection test, developed in collaboration with the Mayo Clinic (Rochester, MN, USA). Although this test is still under development, it already has been granted (November 2019) a Breakthrough Device designation by the FDA. This designation is awarded on the basis of manufacturer request upon documentation of the test performance and allows for faster device development stimulated by the interaction with the FDA as an advisory body. The test detects methylation of a panel of four genes (*DAB2IP*, *EMX1*, *HOXA1*, and *TSPYL5*) and two protein markers (AFP and lectin bound AFP) in blood, with modified QuARTS^TM^ technology and immunochemical techniques, respectively. The initial results describing the performance of the test were presented at The American Association for the Study of Liver Diseases (AASLD) Liver Meeting (Boston, MA, USA, 8–12.11.2019), included 137 patients with HCC and 313 controls and showed the overall panel sensitivity to detect HCC, at the level of 80.3% (95% CI 72.6–86.6) vs. 42.3% (95% CI 33.9–51.1) for AFP 20 ng/ml, with the specificity of 90.0% and 97.4%, respectively [44].

One more very promising test, that also received a Breakthrough Device designation (September 2019), is IvyGene Dx Liver Cancer Test (Laboratory for Advanced Medicine Inc., Irvine, CA, USA). The test analyzes methylation status of genes in cfDNA extracted from plasma, using next-generation sequencing (NGS) approach, to confirm the presence of liver cancer (the manufacturer has not yet provided the information about the genomic localization of specific targets of this assay). The data from preliminary clinical study, presented during the 33rd Annual Meeting of the Society for Immunotherapy of Cancer (SITC) (Washington, DC, USA, 2018) [45], indicated 95% test sensitivity and 97.5% specificity of the test for the detection of hepatocellular carcinoma. The performance of the test is now evaluated in ongoing clinical trial (NCT03694600) comparing the performance of the IvyGene Dx Liver Cancer Test, ultrasound alone, and the combination of IvyGene Dx Liver Cancer Test and ultrasound for the detection of HCC within the patients with liver cirrhosis.

#### Lung cancer

Lung cancer is the leading cause of death from cancer worldwide, responsible for as many deaths as the four most deadly cancers breast, prostate, colon, and pancreas, combined [46].

Epi proLung® (also manufactured by Epigenomics AG, Berlin, Germany) is currently the only CE-marked test that we were able to identify, intended for lung cancer diagnosis in patients at increased risk of the disease. The test detects methylation of *SHOX2* and *PTGER4* genes in the cfDNA extracted from plasma using real-time PCR with a fluorescent hydrolysis probe. The performance of *SHOX2*/*PTGER4* biomarkers to detect lung cancer in plasma samples was initially tested in the study that included 118 lung cancer patients, covering all major histological types and a broad range of stages and 212 healthy controls. The study reported test sensitivity at the level of 67% (at a fixed specificity of 90%) and specificity of 73% (at a fixed sensitivity of 90%) [47]. Subsequent validation study performed on 172 subjects (50 lung cancer; 50 nonmalignant lung diseases, as asthma, chronic obstructive pulmonary disease, or pneumonia; 72 healthy controls) showed that the test allowed differentiation of lung cancer patients from patients displaying symptoms of lung cancer but suffering from non-cancer diseases [47]. Moreover, the results that manufacturer presents in the test user manual that includes 152 subjects with pathologically confirmed lung cancer and 208 subjects not diagnosed with lung cancer report the test sensitivity to detect lung cancer at the level of 59% (at a fixed specificity of 95%) and specificity of 50% (at a fixed sensitivity of 85%), with AUC = 0.82.

#### Bladder cancer

Bladder cancer (BC) is the most common neoplasm of the urinary system [48]. Clinically, 75 to 80% of bladder tumors are diagnosed at stages Ta, T1, and carcinoma in situ (CIS), referred to as non-muscle-invasive bladder cancers (NMIBC) [48], whereas stages T2/3, T4, N^+^, (lymph node metastasis), and M^+^ (metastasis) are referred to as muscle-invasive bladder cancer (MIBC) [49]. Patients treated for NMIBC but progressing to MIBC were shown to have a worse prognosis than patients with primary MIBC, with the 5-year survival rates of 28% (95% CI 15–41) for progressive and 55% (95% CI 43–67) primary muscle-invasive bladder cancer patients [49]. The methylation changes were shown to take part in the progression from primary NMIBC to MIBC [48, 50, 51], what initiated the development of a methylation-based test for the diagnosis of the BC.

We have been able to identify two tests targeting methylation changes in the tumor DNA released to urine for the early detection of BC, AssureMDx (MDxHealth, Irvine, CA, USA) and Bladder *CARE*™ (Pangea Laboratory, Costa Mesa, CA, USA). Both of these tests are laboratory-developed tests (LDT), meaning that the tests are designed, manufactured, and used within a single health institution/laboratory.

AssureMDx test targets methylation of *OTX1*, *ONECUT2*, and *TWIST1* genes in DNA extracted from urine, using multiplex SNaPshot® assay, and mutations in *FGFR3*, *TERT*, and *HRAS* genes using multiplex PCR technology. The test is intended to determine the risk of BC for patients diagnosed with hematuria (blood in urine). The initial validation of AssureMDx was performed in a prospective, multi-center study that included 200 patients (97 with bladder cancer, 103 with non-malignant hematuria), after cystoscopy for microscopic or macroscopic hematuria with no prior history of bladder cancer. This study reported a sensitivity of the test to detect BC at the level of 93%, specificity of 86%, and of 0.96 (95% CI 0.92–0.99) after adjustment for age [52].

Bladder *CARE*™ test is intended for both BC screening and BC recurrence surveillance. The testing procedure is based on digestion of the DNA isolated from urine with a methylation-sensitive restriction enzyme, followed by real-time PCR technology-based methylation detection (MSRE-qPCR; patent application nr WO2016138105A2). The test targets methylation of three genes: *SOX1*, *IRAK3*, and *LINE1*. Initial validation of the test was conducted on a group of 97 BC patients and 85 healthy individuals. The results showed the sensitivity of the test to detect BC at the level of 93.8% and specificity of 85.9% (presented at the 39th Sanford Burnham Prebys Medical Discovery Institute Annual Symposium, La Jolla, CA, USA, 2018).

Worth mentioning here, although still under clinical evaluation, is the bladder cancer detection test named UroMark, developed by the University College London (UK) researchers. The test targets the methylation status of 150 loci (CpG sites) across the genome, using microdroplet-based PCR amplification of bisulfite-converted DNA followed by NGS of the amplified target loci (termed RainDrop BS-Seq) [53]. The initial study indicating utility of this test included 274 patients (167 non-cancer and 107 bladder cancer) and reported the test sensitivity to detect primary BC at the level of 98%, specificity of 97%, and negative predicting value (NPV) of 97% with AUC = 97%, compared to non-BC urine [54]. The test was further evaluated in two multi-center prospective observational studies: DETECT I (Clinical Trial NCT02676180) and DETECT II (Clinical Trial NCT02781428), aiming to assess the performance of the test to rule out bladder cancer in patients with hematuria as well as detect bladder cancer in participants with new or recurrent disease but data from these studies are not yet available.

#### Prostate cancer

Prostate cancer is the most prevalent cancer in men worldwide and the second major cause of cancer death among men [55]. The current standard diagnosis of this cancer is based on histopathological examination of prostate tissue collected via needle biopsy. Due to the specific anatomy of the prostate gland, 60–70% of initial prostate biopsies fail to detect cancer. At the same time, 20–30% of men receive false-negative results [56]. This leads to a high rate of repeat biopsy, which increases health-care costs and potential morbidity, caused by biopsy-related infection, or sepsis [57].

We have not been able to identify CE-marked or FDA-approved tests for early detection of this cancer type but one laboratory-developed test, ConfirmMDx (MDxHealth, Irvine, CA, USA), was shown to be useful in the assessment of the risk of occult disease in men with negative prostate cancer biopsy. The test is built on a multiplexed quantitative DNA methylation-specific PCR technology and targets the methylation status in three genes: *GSTP1*, *RASSF1*, and *APC* in the DNA derived from the prostate core biopsy tissue. The clinical utility of these biomarker panel was investigated in the MATLOC study performed on archived tissue from needle core biopsy of 498 subjects with histopathologically negative prostate biopsy, followed by repeat biopsy within 30 months. When patients with positive repeat biopsy were considered cases and with negative were controls, the sensitivity of the panel to detect prostate cancer was the level of 68% (95% CI 57–77) and specificity 64% (95% CI 59–69) [58]. Further validation of the test was performed in the multicenter study that included 211 African American patients, undergoing 12-core transrectal ultrasound-guided repeat biopsy within 30 months from a cancer-negative biopsy [57]. The study showed 74.1% (95% CI 63.1–83.1) sensitivity and 60.0% (95% CI 51.1–68.5) specificity of the test to detect any prostate cancer in the repeat biopsy, and 77.8% sensitivity (95% CI 57.7–91.4) and 52.7% (95% CI 45.2–60.1) specificity to detect high-grade prostate cancer. Overall, the high NPV of 78.8% (95% CI 71.5–84.6%) of this test indicates its utility to identify men who could potentially avoid a repeat prostate biopsy procedure. The performance of the test is currently assessed in the prospective clinical trial (NCT03082274).

#### Cervical cancer

Cervical cancer is the fourth most common cancer in women worldwide and the fourth most common cause of death from cancer in women [33]. This cancer develops from cervical intraepithelial neoplasia (CIN), a premalignant lesion, histologically graded as CIN1, CIN2, and CIN3 [59]. The risk of progression of CIN1 to invasive cancer is low and most of these lesions undergo spontaneous regression [60]. However, the risk of progression for CIN2 and CIN3 is much higher [61]. Persistent infection with carcinogenic human HPV is necessary for the development of CIN and subsequently cervical cancer [62]. Thus, the prevention of cervical cancer is based on the detection of the CIN and assessment of the oncogenic subtypes of human papillomavirus (HPV).

GynTect® (Oncognostic GmbH, Jena, Germany) is a methylation biomarker-based CE-marked test dedicated to the diagnosis of CIN or cervical cancer in HPV-positive women. It targets methylation of the panel of six genes: *ASTN1*, *DLX1*, *ITGA4*, *RXFP3*, *SOX17*, and *ZNF671*, in the DNA extracted from cervical smear, using methylation-specific real-time PCR technology. The test was shown to detect cervical intraepithelial neoplasia above CIN3 (CIN3^+^) with a sensitivity of 64.8% and specificity of 94.6% [63].

Other CE-marked test for the detection of cervical cancer that we have been able to identify is QIAsure Methylation Test (QIAGEN GmbH, Hilden, Germany), intended for women tested HPV-positive or with cervical smear cytology showing atypical squamous cells of undetermined significance. The test detects the hypermethylation of the *FAM19A4* and *MIR124*-*2* genes in DNA isolated from cervical or vaginal specimens, using multiplex real-time PCR technology. The clinical validation of the test was initially performed on 1680 HPV-positive cervical samples, which originated from seven different European locations. This study showed 67% sensitivity for CIN3 and 100% sensitivity for cervical cancer, in the samples collected in clinician settings which was similar to the sensitivity observed for self-collected samples [64]. The test was further validated in the study including 519 invasive cervical cancer samples from 27 countries. This study reported the hypermethylation of *FAM19A4* and *MIR124*-*2* genes in 98.3% (95% CI 96.7–99.2) of studied samples and the frequency of the biomarkers methylation was consistent regardless of cervical cancer histotype, FIGO stage (following International Federation of Gynecology and Obstetrics classification), HPV genotype, sample type, and geographical region [65].

The iStat Biomedical Co. Ltd. (New Taipei City, Taiwan) is marketing two CE-marked tests for cervical cancer detection: PAX1 DNA Detection Kit and ZNF582 DNA Detection Kit, targeting *PAX1* and *ZNF582* genes, respectively. The tests are built on real-time PCR technology and are intended for the detection of either cervical or oral cancer, in the DNA from cells collected by the scraping. The utility of *PAX1-*based test was initially investigated in the study that included 247 cases with normal and 172 cases with abnormal results of the commonly used cervical Pap test. The results showed the test sensitivity to detect CIN3^+^ at the level of 92% and specificity of 83%, with AUC = 0.97 [66]. The performance of both tests but referenced under the different names, Cervi-P (PAX1 DNA Detection Kit) and Cervi-Z (ZNF582 DNA Detection Kit), was further validated in a study including 449 women referred for colposcopic examination by gynecologists, where methylation of *PAX1* or *ZNF582* in combination with the presence of HPV16/18 was associated with CIN3^+^, OR 15.52, 95% CI 7.73–31.18. Furthermore, the results of this study showed the sensitivity and specificity of Cervi P test combined with HPV16/18 to detect CIN3^+^ at the level of 89.2% (95% CI 83.5–93.2) and 76.0% (95% CI 70.7–80.5), respectively. And sensitivity and specificity of Cervi Z test combined with HPV16/18 was 85.4% (95% CI 79.1–90.1) and 80.1% (95% CI 76.2–85.2), respectively [67].

#### Early detection of cancer regardless of its type

A postulate to screen the general population or population with increased risk for cancer with a simple liquid-based test is a very attractive concept with the benefits including decreasing cancer burden across entire populations and significant health care system savings. With the above goal, GRAIL Inc. (Menlo Park, California, USA) conducts one of the largest programs in genomic medicine that includes three studies: Circulating Cell-free Genome Atlas (CCGA) Study (Clinical Trial NCT02889978), STRIVE Study (Clinical Trial NCT03085888), and SUMMIT Study (Clinical Trial NCT03934866). The test that the company is developing, in May 2019, achieved Breakthrough Device designation.

The CCGA Study enrolled 15254 participants, with ~ 70% of participants being either newly diagnosed with cancer or yet to receive treatment at the time of enrolment. First data from CCGA study were presented at the annual meeting of the American Society of Clinical Oncology (Chicago, IL, USA, 1–5.06.2018), and included results from a cohort of 1785 participants: 984 participants with newly diagnosed, untreated cancer (20 tumor types, all stages) and 749 non-cancer participants. Three prototype NGS-based assays were evaluated in that study including assay one—targeted sequencing of the panel of 507 genes with the aim to identify single nucleotide variants/indels in paired cfDNA and DNA from white blood cell (WBC), assay two—whole-genome sequencing (WGS) to identify copy number variation in paired cfDNA and DNA from WBC, and assay three—whole-genome bisulfite sequencing (WGBS) to characterize methylation in cfDNA of patients and controls. The study reported > 50% sensitivity and 98% specificity, to detect anorectal, triple-negative breast, colorectal, esophageal, head and neck, hepatobiliary, lung, lymphoma, ovarian, and pancreas cancers (the company did not disclose how these parameters were calculated). Importantly, the study concluded that WGBS assay was most accurate, which led GRAIL Inc. to focus only on methylation signatures in subsequent studies [68]. Thus, the second data reported from CCGA Study described the development of the “targeted methylation assay” using Machine Learning Algorithm and data from already existing and new database of DNA methylation profiles of plasma and tissue samples. Specifically, the data used for the assay development included cfDNA methylation sequence data of 4000 samples from cancer participants, along with 1000 matched tissue samples data, cfDNA of 2000 samples from non-cancer participants, and the public sequence data from The Cancer Genome Atlas Oncoviruses [69]. The assay was created not only to differentiate cancer and non-cancer samples but also to detect tissue of origin (TOO) across a set of more than 20 cancer types. Clinical performance of the “targeted methylation assay” was evaluated in another CCGA nested study that included 2301 participants (1422 cancer [> 20 tumor types, all stages], 879 non-cancer). The sensitivity of the assay across all cancer types ranged from 59 to 86% (at 99% specificity), with 34% (95% CI 27–43) sensitivity in stage I (*n* = 151), 77% (95% CI 70–83) in stage II (*n* = 171), 84% (95% CI 79–89) in stage III (*n* = 204), and 92% (95% CI 88–95) in stage IV (*n* = 281). Moreover, the assay assigned tissue of origin to 94% of cancers in the study and for 90% of these cases, the assignment of TOO was correct [70].

The STRIVE and SUMMIT studies are still enrolling, with STRIVE Study focusing on the further development of the targeted methylation test in an asymptomatic population, while the aim of SUMMIT Study is to investigate how the screening test can be improved.

The Laboratory for Advanced Medicine Inc. (Irvine, CA, USA) is also a company developing test aiming to detect multiple cancer types already marketed under the name: IvyGene® Cancer Blood Test. The test is currently available as an LDT test and it targets methylation status *MYO1G* and *TNFAIP8L2* genes, in cfDNA from blood with NGS approach. The initial validation of the test was performed using plasma from 197 subjects with either no history of cancer or a diagnosis of breast, colon, lung, and liver cancer, showing 84% (95% CI 75–93) sensitivity and 90% (95% CI 85–95) specificity to detect these four cancers. However, preliminary results also showed that the positive result of the test may indicate other types of cancers [71]. Therefore, in the current form, the IvyGene® Cancer Blood Test needs to be used in combination with other diagnostic tests, including mammograms, biopsies, or positron emission tomography (PET) scans.

### Disease management biomarkers

Personalized (precision) medicine is built on IVD tests, results of which are used to make medical decisions tailored to the individual patient. The biomarkers in the field of personalized medicine in general terms can be categorized as either prognostic or predictive. The prognostic biomarkers indicate the likelihood of a future clinical event, as, e.g., death, disease progression, development of a new medical condition [72]. The predictive biomarkers allow identification of the individuals who are more likely to respond to a particular medical intervention, e.g., medication, medical devices or procedure, and behavioral or dietary modifications for disease treatment. The response here is defined as “symptomatic benefit, improved survival, or an adverse effect” [73]. To establish the predictive value of a biomarker, only association with clinical outcome needs to be shown. However, to establish biomarkers’ predictive value, at least two independent, randomized clinical trials evaluating the outcome of the intervention and control treatment in individuals with and without the biomarker is needed [74]. With the evidence from a vast number of research studies, there is no doubt that disease-specific methylation changes can be used as both prognostic and predictive biomarkers and can play a significant role in personalized medicine. However, the use of methylation biomarkers in in vitro diagnostics for personalization of patient care is still marginal. This can mainly be attributed to already mentioned general lack of well-designed studies providing data of sufficient quality for the regulatory approvals of the potential IVD test but also lack of the standardization of the methylation testing methods. The urgent need for standardization and subsequent systematic evaluation of the clinical performance of the methylation-based IVD tests in personalized medicine is best exemplified by the fact that we have only been able to identify tests certified for diagnostic use as a predictive test for the management of three indications: glioblastoma multiforme, breast cancer, and cancers of unknown primary site.

#### Glioblastoma

Glioblastoma multiforme (GBM) is the most common primary malignant brain tumor in adults [75]. The median overall survival (OS) of GBM patients after diagnosis ranges from 16 to 21 months [76] and only about 2–3% of patients survive up to 2 years after treatment with the standard therapy, that includes tumor resection followed by radio- and chemotherapy [77].

Standard chemotherapy in glioblastoma is based on alkylating agents such as temozolomide and studies have long shown that the effectiveness of temozolomide therapy depends on the methylation status of the *MGMT* gene. MGMT protein is a key component in the process of repair DNA damage induced by alkylating agents [78]. Thus, tumors with *MGMT* gene promoter methylation and downregulation of the MGMT protein respond to the treatment [79–81]. We have been able to identify three CE-marked and two LDT tests for the detection of *MGMT* methylation status currently available on the market, all developed to detect *MGMT* methylation in DNA extracted from formalin-fixed paraffin-embedded (FFPE) samples.

The *Therascreen*® MGMT Pyro® Kit manufactured by QIAGEN GmbH (Hilden, Germany) is a CE-marked kit, based on Pyrosequencing® technology, and targets four CpG sites in exon 1 (assembly GRCh37/hg19, chr10:131265522–131265539) of the *MGMT* gene. The performance of that test was evaluated in a study that included 48 primary GBM samples, using four meningioma samples as negative controls. The study showed that the *therascreen*® MGMT Pyro® Kit, accurately detects *MGMT* promoter methylation and can be used for patients stratification according to OS (hazard ratio (HR) of 2.3348 (95% CI 1.1918–4.5724; *p* = 0.013) [82].

The Human MGMT Gene Methylation Detection Kit (Multiplex Fluorescence Polymerase Chain Reaction) from Xiamen Spacegen Co., Ltd. (Xiamen, Fujian Province, China), is also CE-marked test and based on PAP-ARMS® technology which is a combination of amplification refractive mutation system (ARMS) [83] and pyrophosphorolysis-activated polymerization reaction (PAP) [84]. The performance of the test was assessed by head to head comparison to Sanger sequencing and among 100 samples included in this study, the test identified 39 and Sanger 34 methylation-positive samples respectively (data from user manual provided by the manufacturer).

The third CE-marked test is MGMT Promoter Methylation Detection Kit from EntroGen Inc. (Los Angeles, CA, USA). The test targets 12 CpG sites in the exon 1 (assembly GRCh37/hg19, chr10:131265519–131265610) of *MGMT* using semi-quantitative real-time PCR with fluorescent hydrolysis probes and methylation-specific primers. According to the manufacturer, the test can be used not only with DNA extracted from FFPE but also from tumor biopsies or fresh frozen glioblastoma tumors. The performance of this test was evaluated in a study that compared the performance of the test to LDT, MGMT methylation test developed by Knight Diagnostic Laboratories (Oregon, PNW, USA), which utilizes pyrosequencing to detect methylation at seven CpG sites in the exon 1 (GRCh37/hg19, chr10:131265514–131265544) of *MGMT* promoter. The results of the comparison were presented at the Association for Molecular Pathology (AMP) Annual Meeting & Expo, Baltimore, USA, November 2019 and the study that included 75 GBM tumor samples and 14 control samples showed that both tests were able to identify all patients with methylated *MGMT* promoter included in the study. The PredictMDx is the second LDT test for *MGMT* methylation detection that we identified. The test is manufactured by LabCorp, Burlington, NC, USA (under the license from MDxHealth, Irvine, CA, USA) and is built on quantitative methylation-specific polymerase chain reaction technology. The most recent reports (Clinical Trial NCT00884741) indicating utility of this test are results from phase III randomized trial of bevacizumab, that included 637 GBM patients. In that trial, the test was able to stratify patients with different progression-free survival (PFS), which for patients with no methylation of *MGMT* gene promoter was 8.2 months and with methylated *MGMT* 14.1 months (HR 1.67 (95% CI 1.36–2.05; *p* < 0.001)) and median OS for patients with unmethylated and methylated *MGMT* gene promoter was 14.3 months and 23.2 months, respectively (HR 2.10 (95% CI 1.65–2.68; *p* < 0.001)), regardless of treatment [85].

#### Breast cancer

Breast cancer is the most common cause of death from cancer in women, with over 2 million new cases worldwide yearly [33] and this cancer is a second disease were we identify CE-marked kit intended to predict patients’ prognosis. The test is marketed under the name *Therascreen*® PITX2 RGQ PCR Kit (QIAGEN GmbH, Hilden, Germany) and targets methylation changes at *PITX2* gene which in several studies have been shown to have prognostic [86, 87] and predictive [88] significance in breast cancer. The test utilizes real-time PCR with fluorescent hydrolysis probes and methylation-unspecific primers to detect methylation of three CpG sites within of *PITX2* gene (assembly GRCh37/hg19, chr4: 111558429–111558431, chr4:111558435–111558437, chr4:111558443–111558445), in the DNA extracted from FFPE breast cancer tissue [89]. The clinical validation of the test was initially performed in the study that included 205 archival FFPE breast cancer samples of lymph node-positive (LN^+^), estrogen receptor-positive (ER^+^), HER2-negative patients (HER2^−^), treated with adjuvant anthracycline-based chemotherapy. This study reported that the test allowed to stratify patients undergoing adjuvant anthracycline-based chemotherapy regarding disease-free survival, with HR 2.74 (95% CI 1.65–3.54; *p* < 0.001) [90].

#### Cancers of unknown primary site

Cancers of unknown primary site (CUP) are the heterogeneous group of cancers for which the tissue of origin remains unknown even after thorough histological and clinical investigation [91]. CUP account for about 3–9% of all cancer diagnoses [91] and with a median age at presentation of 60 years are the fourth most common cause of cancer-related deaths worldwide [92]. For this type of neoplasia, the diagnosis of the origin of the tumor is critical for the choice of the treatment. Developed by Grupo Ferrer Internacional SA (Barcelona, Spain), EPICUP® is a CE-marked test that utilizes bead array technology (Illumina, Inc.) to classify the origin of the CUP. The classifier that the test is using to assign the tumor to the tissue of origin was developed using Infinium® Human Methylation 450K BeadChip (Illumina, Inc.) profiles of 2790 tumors of known origin, representing 38 tumors types and including 85 metastases. The validation of the classifier was performed on a cohort consisting of 7691 known tumors samples from the same tumors types that included 534 metastases. This study reported a 99.6% specificity (95% CI 99.5–99.7) and 97.7% sensitivity (95% CI 96.1–99.2) of the test. The test was able to correctly predict a primary site of cancer for 87% of CUP and patients that received a tumor type-specific therapy, selected according to that prediction, showed improved overall survival compared with patients who received empiric therapy (HR 3.24, *p* = 0.0051 (95% CI 1.42–7.38); log-rank *p* = 0.0029). As Infinium® Human Methylation 450K BeadChip is no longer manufactured, the study also assessed utility of the second generation of the chip Infinium® EPIC Chip to provide accurate classification and concluded that for eight studied CUP cases, the result obtained with new version of the chip were identical [93].

### Post-treatment monitoring/surveillance biomarkers

With the advances in the treatment, an increasing number of diseases especially cancers become chronic diseases and there is a growing need for the molecular tests to evaluate disease recurrence or progression in patients in remission. The tests already described in the section “Diagnostic biomarkers” intended for the disease detection, can potentially be utilized in the detection of the recurrence. However, another clinical study evaluating such a use of the test needs to be performed before different intended use of the test can be proposed. The test targeting methylation biomarkers with the most advanced clinical validation for the use post-treatment monitoring or surveillance that are currently close to be marketed are Colvera^TM^ (Clinical Genomics Pathology Inc, Bridgewater, NJ, USA) and Bladder EpiCheck® (Nucleix Ltd., Rehovot, Israel).

#### Colorectal cancer

The Colvera^TM^ is one of the most advanced in developing post-treatment monitoring test intended to detect recurrence in asymptomatic CRC patients. Currently, the test is provided as an LDT test. The test targets methylation changes of *BCAT1* and *IKZF1* genes in a circulation tumor DNA (ctDNA) with methylation-specific multiplex real-time PCR technology. The utility of the test was initially validated in a study that included 1381 patients scheduled for colonoscopy according to standard guidelines. The results of the study showed the test sensitivity to detect CRC recurrence at the level of 62% (95% CI 49–74) and specificity of 92% (95% CI 90–93). The FIT assay also used in this study showed sensitivity of 79% (95% CI 67–88), but significantly lower specificity (81% (95% CI 78–83)) [94]. The most recent results reporting performance of the test were presented at the American Society of Clinical Oncology (ASCO) annual meeting (June 2019) where the test was compared with the current standard testing for carcino-embryonic antigen (CEA) in plasma, using LIAISON® CEA assay (DiaSorin S.p.A, Saluggia, Italy). The study included 131 patients with relapsed CRC and showed that Colvera™ had a significantly higher sensitivity for detecting CRC recurrence then CEA (68.1% vs. 31.9%; *p* < 0.001) with a similar specificity (97.6% vs. 96.4%; *p* = 0.6547). Additionally, multivariate analysis in that study showed that Colvera™ could independently predict CRC [95]. Currently, Colvera™ performance is evaluated in two observational prospective case-control clinical trials, aiming to compare the performance test to CEA test in 18 years and older. One of these clinical trials (NCT03706248) is already completed and recruited 65 participants, while the other (NCT03706235) is still active with the aim to enroll 550 participants.

#### Bladder cancer

The Bladder EpiCheck® is the second example of the post-treatment monitoring test, available as CE-marked test and intended for detection of NMIBC recurrence in patients previously diagnosed with bladder cancer to reduce the number of follow-up cystoscopies. The test is performed using DNA extracted from urine and targets methylation changes at 15 biomarkers (as described in US9458503B2), with methylation MSRE-qPCR technology (patent application WO2016138105A2). The performance of the test is currently evaluated in two ongoing, multicenter, prospective clinical trials: NCT02647112 (Europe and Israel) and NCT02700464 (USA and Canada). Both trials compare the accuracy of EpiCheck® to detect recurrence of BC to the accuracy of the gold standard cytology and cystoscopy results confirmed by pathology results. The initial results from this trial for the group of 353 patients with the history of urothelial carcinoma (UC), undergoing cystoscopy surveillance at 3-month intervals (adjuvant intravesical therapy allowed), reported overall test sensitivity to detect recurrent UC at the level of 68.2% (95% CI 52.4–81.4), specificity of 88.0% (95% CI 83.9–91.4), and negative predictive value of 95.1% (95% CI 91.9–97.3), with AUC of 0.82 [96]. Also, recently published study that included 243 patients undergoing follow up for previously diagnosed NMIBC showed significantly higher sensitivity of the Bladder EpiCheck test (62.3%; 95% CI 32.6–59.7) in NMIBC detection, compared to cytology (33.3%; 95% CI 22.4–45.7). However, the specificity of the test (86.3%; 95% CI 79.6–91.4) did not reach that shown for standard cytology (98.6%; 95% CI 95.1–99.8). This study concluded that the test can be used in combination with cytology to reduce the invasiveness in the follow-up of NMIBC [97].

## Conclusions

Considering the magnitude of the research that is performed in the field of methylation biomarkers, described in the above IVD applications that we were able to identify are still very limited. This exemplifies urgent need to stimulate the process of application of the methylation biomarker research in clinical use. The translation of the research findings into clinical practice is inevitably dependent on the interest of the industry as a stakeholder. For many years, methylation biomarker diagnostic sector was not able to generate significant returns on the investment. Furthermore, IVD market is very challenging from the regulatory perspective. However, recent successes of the companies such as EXACT Sciences Corporation, currently revolutionizing the colorectal cancer screening with the test that includes methylation biomarkers, hopefully will stimulate the investments in this sector. It is therefore likely that in the near future, we will be witnessing long awaited impact of the methylation biomarker on the precision medicine.

## Data Availability

Not applicable.

## Abbreviations

AFP: Alpha-fetoprotein
ARMS: Amplification refractive mutation system
AUC: Area under the curve
AUROCs: Area under the receiver operating characteristics
BC: Bladder cancer
CCGA: Circulating Cell-free Genome Atlas
CE: *Conformité Européenne*
CEA: Carcinoembryonic antigen
cfDNA: Cell-free DNA
CI: Confidence interval
CIN: Cervical intraepithelial neoplasia
CIS: Carcinoma in situ
CRC: Colorectal cancer
ctDNA: Circulation tumor DNA
CUP: Cancers of unknown primary site
FDA: Food and Drug Administration
FFPE: Formalin-fixed paraffin-embedded
FIT: Fecal immunochemical test
GBM: *Glioblastoma* multiforme
gFOBT: Guanic-based fecal occult blood test
HCC: Hepatocellular carcinoma
HPV: Human papillomavirus
HR: Hazard ratio
iAs: Inorganic arsenic
IVD: In vitro diagnostic
IVDR: In Vitro Diagnostic Regulation
LDT: Laboratory developed test
MIBC: Muscle-invasive bladder cancer
MSRE-qPCR: Methylation-sensitive restriction enzyme procedure followed by real-time PCR technology
NGS: Next-generation sequencing
NMIBC: Non-muscle-invasive bladder cancer
NPV: Negative predicting value
OS: Overall survival
PAP: Pyrophosphorolysis-activated polymerization reaction
Pb: Lead
PET: Positron emission tomography
PFS: Progression-free survival
PM: Particular matter
SE: Standard error
TCE: Trichloroethylene
TNBC: Triple-negative breast cancer
TOO: Tissue of origin
UC: Urothelial carcinoma
WBC: White blood cell
WGBS: Whole-genome bisulfite sequencing
WGS: Whole-genome sequencing
