# Correction to: Current status of development of methylation biomarkers for in vitro diagnostic IVD applications

**DOI:** 10.1186/s13148-020-00902-9

**Published:** 2020-07-14

**Authors:** Olga Taryma-Leśniak, Katarzyna Ewa Sokolowska, Tomasz Kazimierz Wojdacz

**Affiliations:** Independent Clinical Epigenetics Laboratory, Pomeranian Medical University, Unii Lubelskiej 1, 71-252 Szczecin, Poland

**Correction to: Clinical Epigenetics (2020) 12:100**

**https://doi.org/10.1186/s13148-020-00886-6**

After publication of the original article [1], the authors notified that only dr. Tomasz Kazimierz Wojdacz should be marked as corresponding author.

The original article has been updated.
